# Intramuscular versus intravenous epinephrine administration in a pediatric porcine model of cardiopulmonary resuscitation

**DOI:** 10.1016/j.resplu.2024.100769

**Published:** 2024-09-13

**Authors:** Megan O’Reilly, Janice A. Tijssen, Tze-Fun Lee, Marwa Ramsie, Po-Yin Cheung, Georg M. Schmölzer

**Affiliations:** a Centre for the Studies of Asphyxia and Resuscitation, Neonatal Research Unit, Royal Alexandra Hospital, Edmonton, Alberta, Canada; b Department of Pediatrics, Faculty of Medicine and Dentistry, University of Alberta, Edmonton, Alberta, Canada; c Department of Paediatrics, Schulich School of Medicine & Dentistry, Western University, London, ON, Canada

**Keywords:** Infant, Pediatric, Epinephrine, Adrenaline, Chest Compression, Resuscitation

## Abstract

**Background:**

American Heart Association Pediatric Life Support guidelines recommend epinephrine administration via intravenous (IV) or intraosseous (IO) route, with endotracheal (ET) administration admissible in the absence of IV/IO access. Establishing IV/IO/ET access can take several minutes and may require proficient skills and/or specific equipment, which may not be readily available in all situations. Alternatively, intramuscular (IM) epinephrine could be administered immediately. At present, there is limited data on the use of IM epinephrine in pediatric resuscitation.

**Aim:**

To compare IM with IV epinephrine in a pediatric porcine model of asphyxia-induced cardiac arrest. We hypothesized that in a pediatric animal model of cardiac arrest, IM epinephrine would result in a similar time to achieve return of spontaneous circulation (ROSC) to IV epinephrine.

**Methods:**

Twenty pediatric piglets (5–10 days old) were anesthetized and asphyxiated by clamping the endotracheal tube. Piglets were randomized to IM or IV epinephrine with bradycardic or asystolic cardiac arrest (*n* = 5/group) and were resuscitated. Time to ROSC was recorded; blood plasma was collected throughout resuscitation for measurement of epinephrine concentration; heart rate, arterial blood pressure, carotid blood flow, cardiac function, and cerebral oxygenation were continuously recorded throughout the experiment.

**Results:**

Time to ROSC and the number of piglets that achieved ROSC were comparable between IM and IV epinephrine groups with either bradycardic or asystolic cardiac arrest.

**Conclusions:**

In a pediatric piglet model of bradycardic and asystolic cardiac arrest, administration of IM epinephrine resulted in similar resuscitative outcomes to IV epinephrine. Although immediate IM epinephrine injection may provide a first-line treatment option until subsequent IV/IO access is established, large, randomized trials are needed to confirm our finding before it can be used during pediatric resuscitation.

## Introduction

Survival to hospital discharge following in-hospital cardiac arrest in pediatric patients is approximately 40–50%, however this is dramatically reduced (5–17%) if the cardiac arrest occurs in an out-of-hospital setting.^1^ The current American Heart Association Pediatric Life Support guidelines recommend the use of the cardiac agent epinephrine, to be administered via intravenous (IV) or intraosseous (IO) route at a dose of 0.01 mg/kg every 3–5 min up to a maximum dose of 1 mg.^1^ Endotracheal (ET) administration may be considered in the absence of IV/IO access, at a dose of 0.1 mg/kg.^1^ In pediatric patients, respiratory arrest is the leading cause of cardiac arrest, usually resulting in a nonshockable rhythm.^1^ In these cases, the earlier epinephrine is administered after cardiopulmonary resuscitation (CPR) initiation, the more likely the patient is to survive.^1^, ^2^, ^3^ Despite this, establishing IV/IO/ET access can take several minutes and may require proficient skills and/or specific equipment, which may not be readily available in all situations. Alternatively, intramuscular (IM) epinephrine could be administered immediately, requiring only standard equipment/resources that are commonly available and a limited skill/training level to administer.

IM epinephrine is universally used as the first-line treatment for anaphylaxis, with current standard practice using a maximum dose of 0.3 mg for pediatric patients weighing up to 30 kg.^4^ Consideration for its use in pediatric, and even neonatal, resuscitation is possible. In a pediatric porcine model (approximately 2–5 weeks of age), IM epinephrine (via the tongue muscle) was shown to promote return of spontaneous circulation (ROSC) after ropivacaine-induced cardiac arrest, displaying comparable survival to piglets administered IV epinephrine.^5^ Similar, a before-and-after study of implementation of an early, first-dose IM epinephrine administration by EMS for adult out-of-hospital cardiac arrest reported that an initial IM dose of epinephrine as an adjunct to standard care was associated with improved survival to hospital admission, survival to hospital discharge, and functional survival.^6^ However, there is limited data regarding absorption and pharmacokinetics of IM administration in the context of pediatric resuscitation. In a case series of four neonatal lambs, IM epinephrine (via the deltoid muscle) was administered 30 sec after CPR initiation; no notable increase in plasma epinephrine concentrations were reported,^7^ although delayed absorption was observed 5 min after injection. Indeed, the bioavailability of IM epinephrine may be limited in scenarios of severe acidosis or asystole, and chest compressions alone may prove insufficient in achieving adequate muscle perfusion for circulation of epinephrine injected into the muscle.^8^ Furthermore, bioavailability of epinephrine after IM injection has been shown to be dependent on the muscle injected, with a peak level five-times higher following vastus lateralis IM injection compared to deltoid muscle.^8^

We aimed to compare resuscitative outcomes in pediatric piglets following asphyxia-induced cardiac arrest and administration of IM or IV epinephrine. We hypothesized that in a pediatric animal model of cardiac arrest IM epinephrine would result in a similar time to achieve ROSC to IV epinephrine.

## Methods

**Fig. 1:**
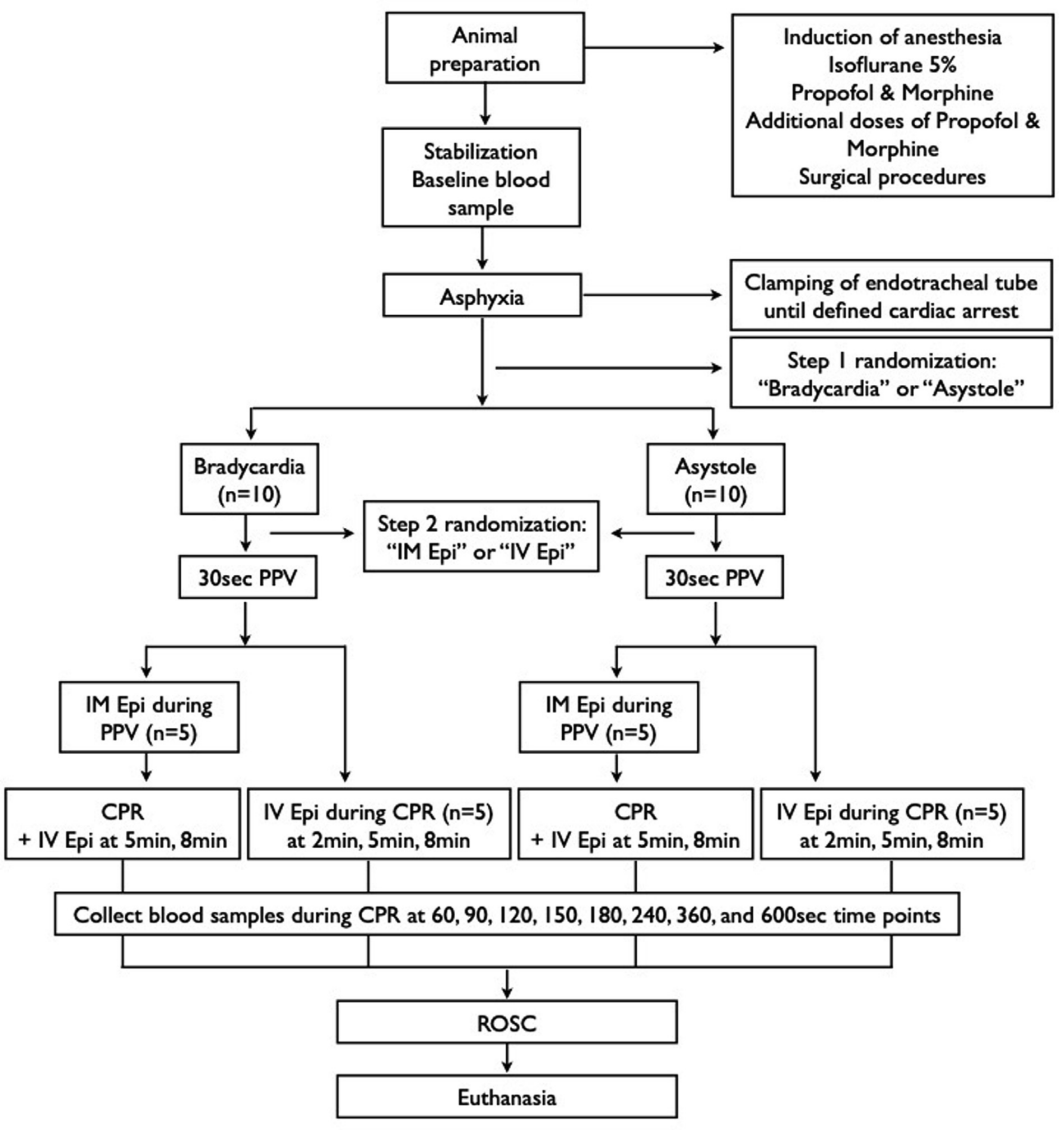
Study flow chart.

Twenty mixed breed pediatric piglets were obtained on the day of experimentation from the University Swine Research Technology Centre located in Edmonton, Alberta, Canada. All experiments were conducted in accordance with the guidelines and approval of the Animal Care and Use Committee (Health Sciences), University of Alberta (AUP4015) and according to the Canadian Council of Animal Care guidelines. Experiments were conducted and presented according to the ARRIVE guidelines^9^ and registered at preclinicaltrials.eu (PCTE0000470). A graphical display of the study protocol is presented in Fig. 1.

### Inclusion and exclusion criteria

Mixed breed pediatric piglets with a current age of 5–10 days old (weighing 2.1–4.3 kg), equivalent to infants 25–35 days old, were included. There was no exclusion criterion.

### Randomization

Twenty piglets were subjected to a two-step randomization process and were randomly allocated to “bradycardia” or “asystole” cardiac arrest (step 1), followed by randomization to “IM” or “IV” epinephrine (step 2). Randomization was 1:1 using a computer-generated randomization program (https://www.randomizer.org). A numbered, sealed, brown envelope was opened at each step containing the group allocation.

### Sample size and power estimates

Our primary outcome measure was the CPR time to achieve ROSC. Our previous studies showed a mean (SD) time of 458 (1 4 0) seconds to achieve ROSC during CPR with bradycardia using IV epinephrine.^10^, ^11^ Based on this, a sample size of 5 piglets per group was sufficient to detect a clinically important (20%) reduction in time to achieve ROSC during CPR with bradycardia, with 80% power and a 2-tailed alpha error of 0.05.^12^ The same sample size (5 per group) was used to examine CPR time to achieve ROSC during asystolic cardiac arrest.

### Blinding

It was impossible to completely blind the team to the allocated interventions due to the nature of providing each intervention. However, the two-step randomization process enabled the concealment of the epinephrine intervention (IM vs IV) until bradycardic or asystolic cardiac arrest was confirmed (by GMS). The statistical analysis was blinded to group allocation and only unblinded after the statistical analysis was completed.

### Animal Preparation

Piglets were instrumented as previously described with modifications.^10^, ^13^, ^14^ Following the induction of anaesthesia using isoflurane, piglets were intubated via a tracheostomy, and pressure-controlled ventilation (Sechrist Infant Ventilator Model IV-100; Sechrist Industries, Anaheim, California) was commenced at a respiratory rate of 16–20 breaths/min and pressures of 20/5 cmH_2_O. Oxygen saturation was kept within 90–100%, glucose level and hydration were maintained with an intravenous infusion of 5% dextrose at 10 mL/kg/hr. During the experiment anaesthesia was maintained with intravenous propofol 5–10 mg/kg/hr and morphine 0.1 mg/kg/hr. Additional doses of propofol (1–2 mg/kg) and morphine (0.05–0.1 mg/kg) were also given as needed. The piglet’s body temperature was maintained within a normal porcine reference range of 38.5–39.5 °C using an overhead warmer and a heating pad.^10^, ^13^, ^14^

### Hemodynamic parameters

A 5-French Argyle® (Klein-Baker Medical Inc. San Antonio, TX) double-lumen catheter was inserted via the right femoral vein for administration of fluids and medications. A 5-French Argyle® single-lumen catheter was inserted above the right renal artery via the femoral artery for continuous arterial blood pressure monitoring in addition to arterial blood gas measurements. The right common carotid artery was also exposed and encircled with a real-time ultrasonic flow probe (2 mm; Transonic Systems Inc., Ithica, NY) to measure carotid blood flow.^10^, ^13^, ^14^ A Millar® catheter (MPVS Ultra1, ADInstruments, Houston, TX) was inserted into the left ventricle via the left common carotid artery for continuous measurement of stroke volume, ejection fraction, end-diastolic and −systolic volumes, left ventricular pressure, and left ventricular contractile function (dp/dt_max_, dp/dt_min_).

Piglets were placed in supine position and allowed to recover from surgical instrumentation until baseline hemodynamic measures were stable (minimum of one hour). Ventilator rate was adjusted to keep the partial arterial CO_2_ between 35–45 mmHg as determined by periodic arterial blood gas analysis. Mean systemic arterial pressure, systemic systolic arterial pressure, heart rate, and percutaneous oxygen saturation were continuously measured and recorded throughout the experiment with a Hewlett Packard 78833B monitor (Hewlett Packard Co., Palo Alto, CA).^10^, ^13^, ^14^

### Cerebral oxygenation

Cerebral oxygenation (crSO_2_) was measured using the Invos^TM^ Cerebral/Somatic Oximeter Monitor (Invos 5100, Somanetics Corp., Troy, MI). The sensors were placed on the right forehead of the piglet and secured with wrap and tape. Light shielding was achieved with a slim cap. The Invos^TM^ Cerebral/Somatic Oximeter Monitor calculates crSO_2_, which is expressed as the percentage of oxygenated haemoglobin (oxygenated haemoglobin/total haemoglobin). Values of regional oxygen saturation are stored every second with a sample rate of 0.13 Hz.^15^, ^16^

### Mechanical chest compression machine

We used a custom-designed mechanical chest compression machine for mechanical chest compression delivery.^17^, ^18^, ^19^, ^20^, ^21^ The chest compression machine delivers various compression rates (50–200 per minute), anterior-posterior (AP) chest compression depths (10–70%), acceleration of compressions (100–1000 cm/s^2^), recoil speed (1–100 cm/s), steps per revolution (400–1,200 steps per revolution), and varying duty cycle.

### Experimental protocol

A baseline blood sample was collected prior to asphyxia (=time point 0 sec). Asphyxia was induced in all piglets by disconnecting the ventilator and clamping the endotracheal tube until either bradycardic or asystolic cardiac arrest was achieved (depending on group allocation determined by randomization − step 1; Fig. 1). A numbered, sealed, brown envelope containing the allocation “bradycardia” or “asystole” was opened after clamping the endotracheal tube. Bradycardic cardiac arrest was defined as mean arterial blood pressure <20 mmHg and bradycardia, with a heart rate <60/min.^10^, ^11^, ^22^ Asystolic cardiac arrest was defined as zero arterial blood flow and no audible heartbeat during auscultation. Once cardiac arrest was confirmed, a second numbered, sealed brown envelope containing the intervention allocation “IM” or “IV” epinephrine was opened (step 2 randomization; Fig. 1).

### Interventions

Thirty seconds after cardiac arrest was diagnosed, positive pressure ventilation was performed for 30 sec using a self-inflating bag with 100% oxygen. After 30 sec of positive pressure ventilation, CPR commenced. All piglets received the same CPR. During CPR, chest compressions were mechanically delivered using an automated chest compression machine^23^, ^17^, ^18^, ^19^, ^20^ and asynchronous ventilations were delivered using a self-inflating bag (Laerdel, Stavanger, Norway), with 30 inflations per minute aiming to deliver a peak inflating pressure of 30cmH_2_O, measured with an attached respiratory function monitor (NM3, Respironics, Philips, Andover, MA).^24^, ^25^ The following were the settings of the automated chest compression machine: compression rate of 100/min, acceleration of compression 500 cm/s^2^, speed of recoil of 50 cm/s, a simulated two-thumb technique, and an anterior-posterior depth of 33%. Supplemental oxygen was 100% during CPR. CPR was continued for a maximum time of 11 min. Piglets that were randomized to “IM” were administered one dose of epinephrine (0.3 mg) intramuscularly in the left outer thigh muscle during positive pressure ventilation, prior to the commencement of chest compressions. Thereafter, “IM” piglets received subsequent doses of epinephrine (0.02 mg/kg per dose) intravenously at 5 min and 8 min unless ROSC occurred. Piglets that were randomized to “IV” received their first dose of epinephrine (0.02 mg/kg per dose) intravenously 2 min after the start of positive pressure ventilation. Subsequent doses of epinephrine (0.02 mg/kg) were given intravenously every 3 min until ROSC (i.e., at 5 min and 8 min). Bolus Ringer’s solution (3 mL) was given immediately after each dose of IV epinephrine. ROSC was defined as an unassisted heart rate > 100/min for 15 sec and a diastolic blood pressure of at least 20 mmHg, detected by femoral intra-arterial monitoring and ECG. Blood samples were collected throughout CPR at 60, 90, 120, 150, 180, 240, 360, and 600 sec time points. At the end of the experiment, piglets were euthanized with an intravenous overdose of sodium pentobarbital (100 mg/kg). IM injection site was assessed for tissue damage, including the presence of hematoma, bleeding, or cyst.

### Data collection and analysis

Demographics of study piglets were recorded. Transonic flow probes, heart rate and pressure transducer outputs were digitized and recorded with LabChart® programming software (ADInstruments, Houston, TX). Epinephrine concentration in blood plasma samples was quantified using a commercially available ELISA kit (KA1882, Abnova Corporation, Taiwan) according to the manufacturer’s instructions.

The data was tested for normality (Shapiro-Wilk and Kolmogorov-Smirnov test) and compared using Student’s *t-test* for parametric and Mann-Whitney *U* test for nonparametric comparisons of continuous variables, and Fisher’s exact test for categorical variables. The data are presented as mean (standard deviation – SD) for normally distributed continuous variables and median (interquartile range − IQR) when the distribution was skewed. *P*-values are 2-sided and *p* < 0.05 was considered statistically significant. Statistical analyses were performed with SigmaPlot (Systat Software Inc, San Jose, USA).

## Results

Twenty pediatric mixed breed piglets obtained on the day of the experiment were randomized to receive IM or IV epinephrine with either bradycardic cardiac arrest (*n* = 10) or asystolic cardiac arrest (*n* = 10) (Fig. 1). Baseline characteristics of all groups are presented in Table 1. There was no tissue damage at the IM injection sites.

**Table 1:** Baseline characteristics.

	**Bradycardia**			**Asystole**		
	**IV** (*n* = 5)	**IM** (*n* = 5)	**p-value**	**IV** (*n* = 5)	**IM** (*n* = 5)	**p-value**
Age (days)	10 (8–10)	10 (8–10)	1.000	7 (7–8)	8 (7–10)	0.367
Weight (kg)	3.9 (3.3–4.9)	3.7 (3.4–4.5)	0.511	3.1 (2.6–3.3)	3.3 (3.2–3.7)	0.096
Gender (male/female)	0/5	1/4	1.000	2/3	3/2	1.000
Heart rate (bpm)	200 (176–209)	215 (156–242)	0.690	201 (167–225)	188 (166–218)	0.841
MAP (mmHg)	70 (69–74)	72 (66–74)	0.841	66 (59–75)	65 (60–66)	0.421
Carotid flow (mL/min)	98 (87–133)	117 (92–126)	0.548	102 (76–105)	108 (97–121)	0.222
Cerebral oxygenation (%)	49 (37–55)	41 (39–50)	0.841	37 (32–42)	37 (33–39)	0.841
Stroke volume (mL/min)	3.6 (2.7–4.3)	4.1 (3.4–4.4)	0.413	3.3 (2.6–4.4)	2.9 (2.1–4.1)	0.421
Ejection fraction (%)	38 (27–45)	38 (37–52)	0.730	39 (27–58)	35 (26–60)	0.841
dp/dt max (mmHg)	3391 (2549–4504)	3719 (3366–3864)	0.730	3198 (2723–3943)	3678 (2683–5289)	0.421
dp/dt min (mmHg)	−4534 (−6508 ∼ -3621)	−3932 (−4881 ∼ -3531)	0.556	−4884 (−4949 ∼ -3740)	−4449 (−6436 ∼ -3541)	0.841
pH	7.39 (7.38–7.47)	7.45 (7.34–7.49)	0.917	7.39 (7.34–7.41)	7.42 (7.36–7.45)	0.310
paCO_2_ (torr)	40 (34–41)	33 (32–45)	0.690	39 (38–42)	38 (37–44)	0.310
paO_2_ (torr)	79 (70–82)	80 (77–84)	0.548	72 (68–85)	61 (49–82)	0.421
Base excess (mmol/L)	−1.1 (−1.4 ∼ 1.1)	−0.2 (−2.6 ∼ 1.8)	0.975	−1.7 (−3.7 ∼ -0.3)	0.1 (−1.6 ∼ 1.8)	0.151
Lactate (mmol/L)	2.3 (2.2–2.5)	2.8 (1.8–3.5)	0.421	2.9 (2.5–3.5)	2.8 (2.3–3.7)	0.841

Data are presented as median (IQR); MAP – mean arterial blood pressure, paCO_2_ – partial pressure of arterial carbon dioxide, paO_2_ – partial pressure of arterial oxygen.

### Resuscitation and primary outcome

Median (IQR) time of asphyxia from endotracheal tube occlusion to bradycardia was 552 (277–582)sec and 554 (517–699)sec in IV and IM epinephrine groups, respectively (Table 2, *p* = 0.195). Median (IQR) asphyxial time to asystole was 600 (522–846)sec and 640 (610–785)sec in IV and IM epinephrine groups, respectively (Table 2, *p* = 0.839). At the end of asphyxia in bradycardic piglets, all had sinus bradycardia (median (IQR) heart rate of 58 (52–61) and 51 (41–64) in IV and IM epinephrine groups, respectively, *p* = 0.475) and a mean arterial blood pressure of 16 (13–20) and 17 (14–20) in IV and IM epinephrine groups, respectively (Table 2, *p* = 0.747).

**Table 2:** Characteristics of asphyxia and resuscitation of asphyxiated piglets.

**Bradycardia**	**IV** (*n* = 5)	**IM** (*n* = 5)	**p-value**
Asphyxia time (sec) ^†^	552 (277–582)	554 (517–699)	0.195
Heart Rate (bpm) ^†^ before CPR	58 (52–61)	51 (41–64)	0.475
Mean arterial pressure (mmHg) ^†^ before CPR	16 (13–20)	17 (14–20)	0.747
Achieving ROSC	3 (60)	3 (60)	1.000
CPR time (sec) ^†^	351 (208–600)	600 (110–620)	0.905
ROSC time (sec) ^†^	240 (175–351)	115 (105–640)	0.513
**Asystole**	**IV** (*n* = 5)	**IM** (*n* = 5)	**p-value**

Asphyxia time (sec) ^†^	600 (522–846)	640 (610–785)	0.839
Achieving ROSC	1 (20)	2 (40)	1.000
CPR time (sec) ^†^	600 (390–600)	600 (205–600)	0.690
ROSC time (sec) ^†^	180	205 (180–230)	−

Data are presented as n (%), unless indicated ^†^median (IQR). Achieving ROSC – the number of animals that achieved return of spontaneous circulation during resuscitation; CPR time – the duration of time cardiopulmonary resuscitation was performed; ROSC time – the duration of time required to achieve return of spontaneous circulation.

**Fig. 2:**
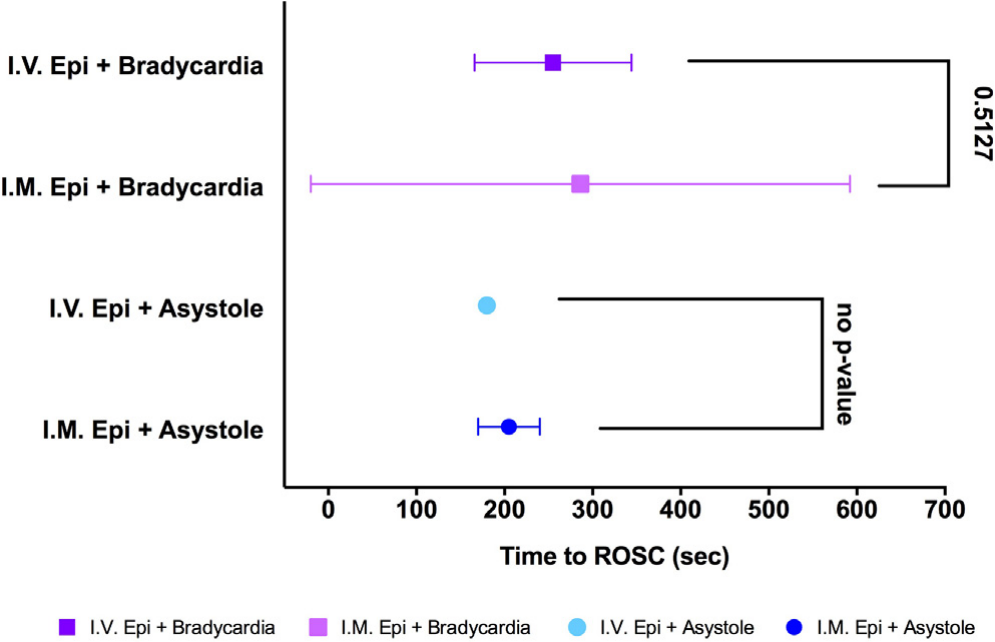
**Time to return of spontaneous circulation.** Time to return of spontaneous circulation (ROSC) in animals that achieved ROSC. Data points are mean (SD). No p-value presented for comparison between asystole groups due to *n* = 1 that achieved ROSC in IV Epi + Asystole group.

The rate of ROSC was not different for IV compared to IM epinephrine in bradycardic piglets 3/5(60%) vs 3/5(60%), respectively (*p* = 1.000). In asystolic piglets the rate of ROSC was not significantly different between IV and IM epinephrine groups, 1/5(20%) vs 2/5(40%), respectively (Table 2, *p* = 1.000). The median (IQR) time to ROSC with IV compared to IM epinephrine in bradycardic piglets was 240 (175–351)sec vs 115 (105–640)sec, respectively (*p* = 0.513), and in asystolic piglets was 180sec vs 205 (180–230)sec, respectively (no p-value or IQR for IV asystolic group due to *n* = 1 achieving ROSC; Table 2^,^ Fig. 2). The epinephrine concentration over time plotted for each individual animal within the IM and IV groups with time to ROSC is presented in a supplemental figure.

### Changes in epinephrine plasma concentrations

**Fig. 3:**
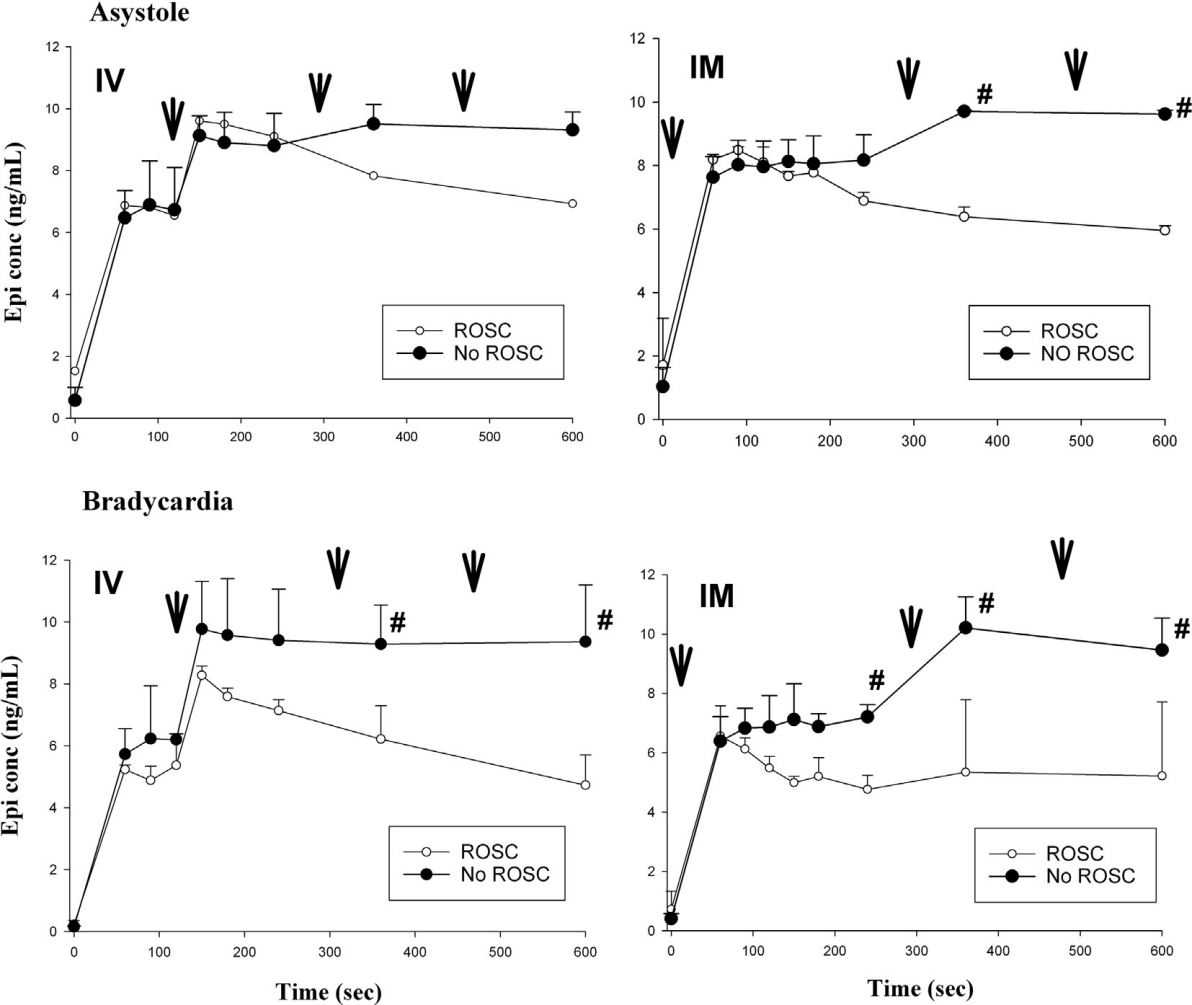
**Epinephrine concentrations in blood samples.** Concentration of epinephrine in blood samples taken at time point 0 sec (baseline), and at 60, 90, 120, 150, 180, 240, 360, and 600 sec of resuscitation. Each point represents mean (SD). Arrows indicate time of epinephrine injection. # indicates significant difference at time point between animals that achieved ROSC and no ROSC (*p* < 0.05; Tukey).

The changes in concentration of epinephrine in blood plasma samples taken over the resuscitation period in bradycardic and asystolic piglets is presented in Fig. 3. Piglets that achieved ROSC sooner required fewer epinephrine doses and thus presented with reductions in their blood plasma concentrations over time. When piglets were exposed to hypoxia and asphyxia, there is an increase in endogenous epinephrine to ∼6 ng/mL (Fig. 3, IV graphs), which further increases after epinephrine administration (Fig. 3). There was no difference in epinephrine concentration between IV and IM concentration regardless of bradycardia (*p* = 0.570) or asystole (*p* = 0.283).

### Changes in hemodynamic parameters

Hemodynamic parameters including heart rate, mean arterial blood pressure, carotid blood flow, and cerebral oxygenation were similar at baseline between IV and IM groups subjected to bradycardia and asystole (Table 1). Hemodynamic changes during resuscitation following bradycardic and asystolic cardiac arrest are presented in Fig. 4a, Fig. 4b, respectively. There were no statistically significant differences between IV and IM groups subjected to bradycardia (Fig. 4a). No p-value is presented for comparison between IV and IM groups subjected to asystole due to *n* = 1 that achieved ROSC in the IV asystolic group (Fig. 4b).

**Fig. 4a:**
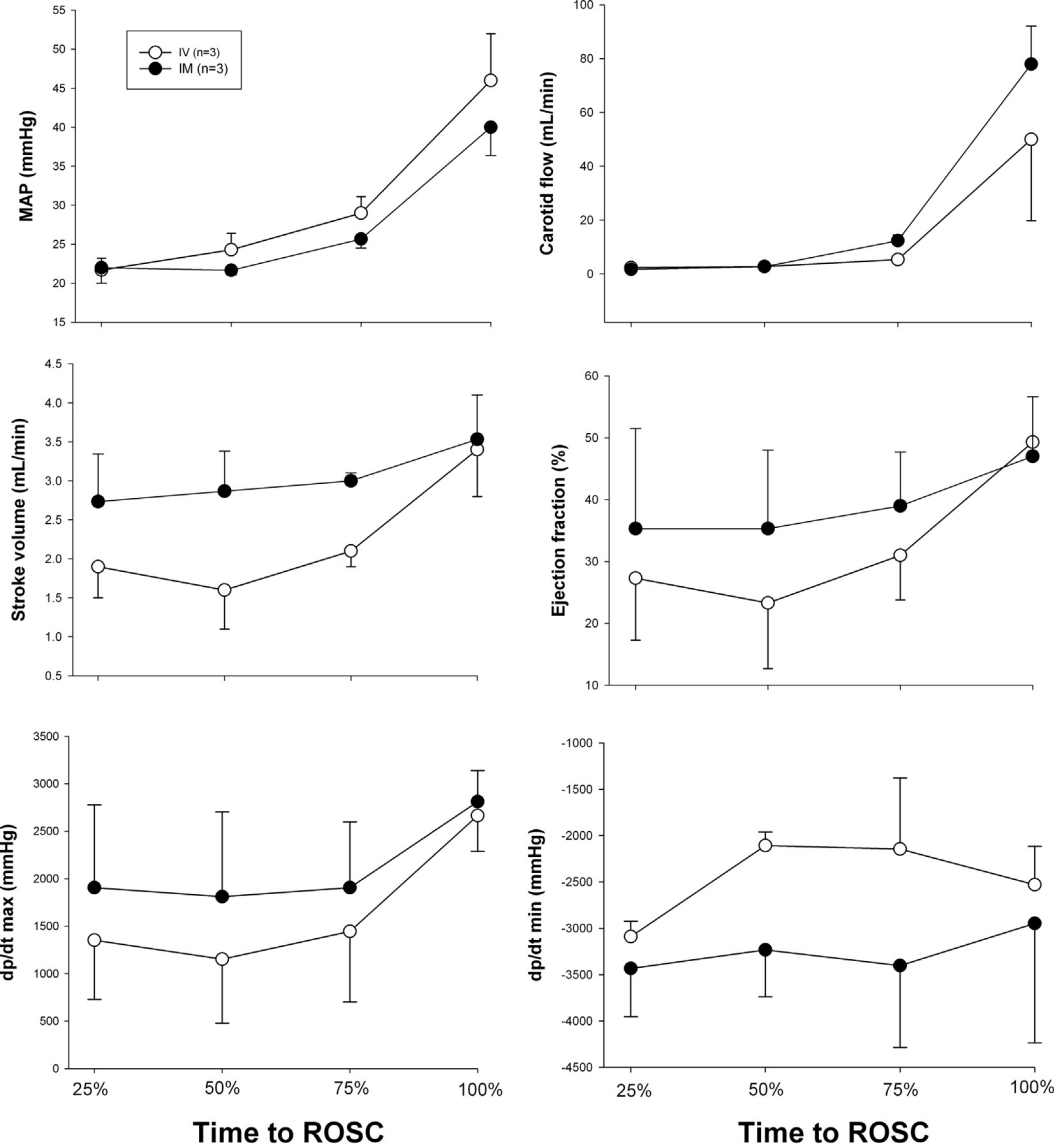
**Hemodynamic changes during resuscitation following bradycardic cardiac arrest.** Hemodynamic changes in MAP – mean arterial pressure (mmHg), carotid blood flow (mL/min), stroke volume (mL/min), ejection fraction (%), dp/dt max and dp/dt min – maximum and minimum rate of left ventricle pressure change (mmHg), respectively. Data are presented as mean (SD) and hemodynamic parameters are plotted against time to ROSC, expressed as a percentage of total time to achieve ROSC. There were no statistically significant differences in hemodynamic parameters between IM and IV groups.

**Fig. 4b:**
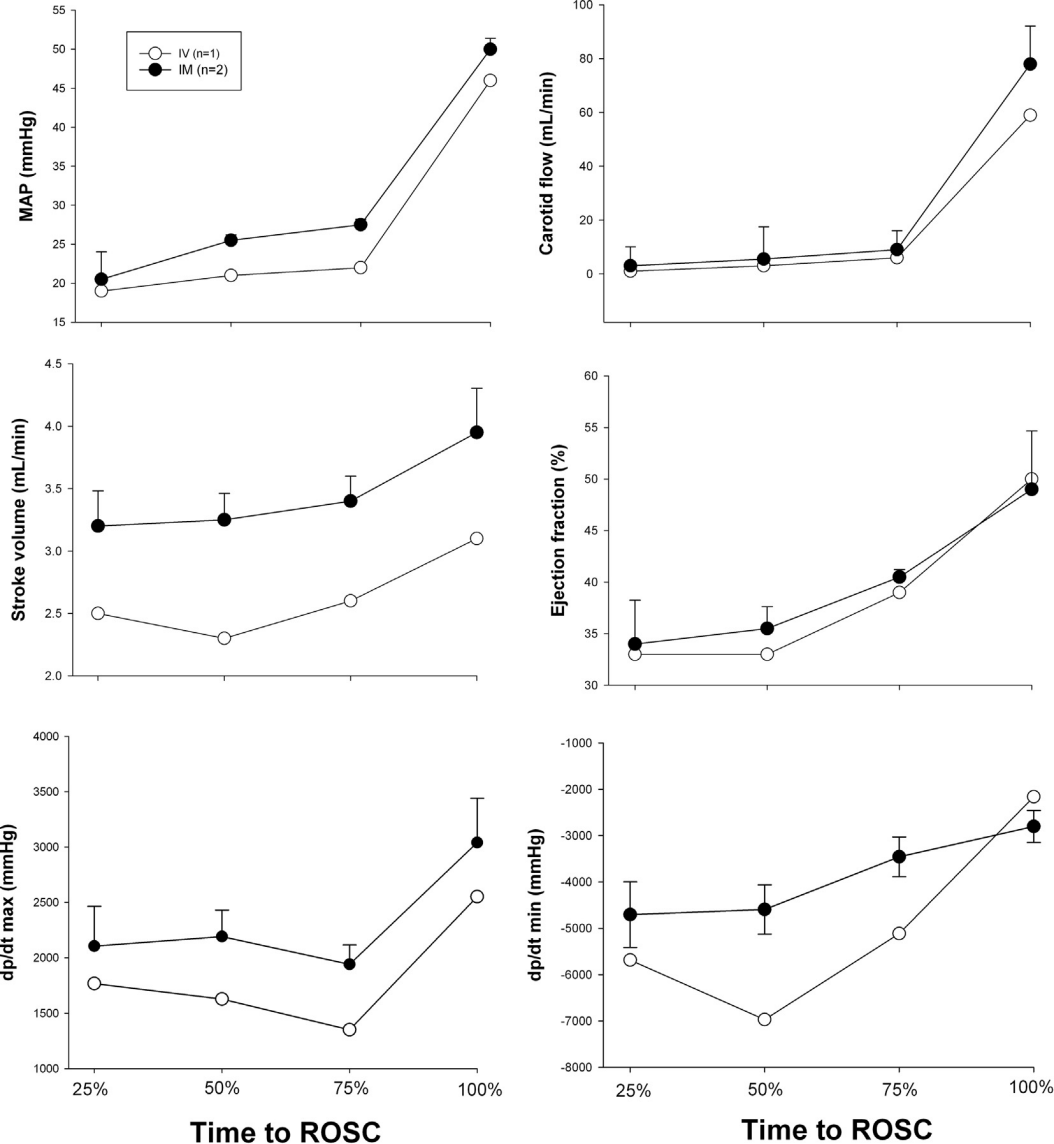
**Hemodynamic changes during resuscitation following asystolic cardiac arrest.** Hemodynamic changes in MAP – mean arterial pressure (mmHg), carotid blood flow (mL/min), stroke volume (mL/min), ejection fraction (%), dp/dt max and dp/dt min – maximum and minimum rate of left ventricle pressure change (mmHg), respectively. Data are presented as mean (SD) and hemodynamic parameters are plotted against time to ROSC, expressed as a percentage of total time to achieve ROSC. No p-value presented for comparison between groups due to *n* = 1 that achieved ROSC in IV group.

## Discussion

Current guidelines for use of epinephrine during pediatric CPR recommend administration via IV or IO access points.^1^ However, in cases of asphyxia resulting in nonshockable cardiac arrest, the time required to receive an initial dose of epinephrine can be a determining factor for subsequent survival. Preparation of access points for administration of epinephrine may take several minutes, owing to a combination of skill level/training required as well as requirement of specialized equipment that may not be readily available in some settings and competing priorities. Given that IM epinephrine is the first line of treatment for anaphylaxis, its use during CPR should be considered. The current study compared resuscitative outcomes of IM and IV epinephrine in a pediatric porcine model of asphyxia-induced bradycardic and asystolic cardiac arrest. The results can be summarized as follows: survival and time to ROSC are comparable between IM and IV epinephrine groups in the setting of bradycardia as well as asystolic cardiac arrest.

Mauch *et al* reported on IM versus IV epinephrine use in a pediatric porcine model utilizing ropivacaine to induce cardiac arrest.^5^ Piglets (aged 2–5 weeks old) were given IM epinephrine in the tongue muscle at a dose of 0.1 mg/kg at 1 min after onset of cardiac arrest; a second dose was administered after 10 min if necessary. Piglets administered IV epinephrine received a dose of 0.01 mg/kg at 1 min after onset of cardiac arrest and then every 4 min as necessary. Mauch *et al* showed that IM epinephrine provided similar survival compared to IV epinephrine.^5^ Similarly, in the current study we show that in an asphyxia-induced model of bradycardic and asystolic cardiac arrest, IM epinephrine provided similar survival and time to ROSC compared to IV epinephrine. Our dose of IM epinephrine, 0.3 mg, was extrapolated from recommendations for anaphylaxis and represents a similar per kg dose to that reported by Mauch *et al*.^5^ In the current study, IM epinephrine was administered during the initial 30 sec of ventilatory resuscitation, prior to initiation of chest compressions. Piglets in the IM epinephrine group also received IV epinephrine (0.02 mg/kg per dose) at 5 min and 8 min unless ROSC was observed. In bradycardic piglets, 2/5 required only IM epinephrine to achieve ROSC, whereas 1/5 required additional IV epinephrine at 5 min and 8 min to achieve ROSC; 2/5 piglets did not achieve ROSC. In asystolic piglets, 2/5 required only IM epinephrine to achieve ROSC, whereas the remaining 3/5 did not achieve ROSC. Piglets in the IV epinephrine group showed similar survival, even though their initial dose was administered later, at 2 min after the onset of bradycardic or asystolic cardiac arrest. This delay to IV epinephrine was shorter than the delay to IV epinephrine in human pediatric cardiac arrest, which is often more than 5 min after paramedic arrival (therefore at least 10 min following cardiac arrest).^26^

In the current study we administered IM epinephrine via injection into the left outer thigh muscle, as this has been demonstrated to generate peak serum epinephrine concentrations five times higher than injection into the deltoid muscle.^8^ This may be indication for the lack of increase in plasma epinephrine in the study by Berkelhamer *et al* following IM injection into the deltoid muscle.

### Limitations

Our piglet asphyxia model closely simulates asphyxia events in young children leading to bradycardia and cardiac arrest, in contrast to models of cardiac arrest induced by other means including ventricular fibrillation. However, several limitations should be considered: All piglets were sedated/anesthetized and intubated with a tightly sealed endotracheal tube to prevent any endotracheal tube leak, which may not occur in all pediatric patients. Nevertheless, our findings are still clinically relevant as the distribution of cardiac output during asphyxia episodes are qualitatively similar. Although we blinded the cardiac arrest assessor, we were unable to blind the intervention due to the difference in administering IM versus IV epinephrine. Although a sample size calculation was performed for this study, we were unable to detect a clinically meaningful difference in time to ROSC between the groups, which may indicate that the study was underpowered.

## Conclusions

In a pediatric piglet model of bradycardic and asystolic cardiac arrest, administration of IM epinephrine resulted in similar resuscitative outcomes to IV epinephrine. Although immediate IM epinephrine injection may provide a first-line treatment option until subsequent IV/IO access is established, large, randomized trials are needed to confirm our finding before it can be used during pediatric resuscitation.

## CRediT authorship contribution statement

**Megan O’Reilly:** Writing – review & editing, Writing – original draft, Validation, Software, Resources, Project administration, Methodology, Investigation, Formal analysis, Data curation, Conceptualization. **Janice A. Tijssen:** Writing – review & editing, Methodology, Investigation, Formal analysis, Conceptualization. **Tze-Fun Lee:** Writing – review & editing, Methodology, Investigation, Formal analysis, Data curation, Conceptualization. **Marwa Ramsie:** Writing – review & editing, Validation, Methodology, Investigation, Formal analysis, Data curation. **Po-Yin Cheung:** Writing – review & editing, Validation, Resources, Project administration, Methodology, Investigation, Formal analysis, Data curation, Conceptualization. **Georg M. Schmölzer:** Writing – review & editing, Validation, Supervision, Resources, Project administration, Methodology, Investigation, Formal analysis, Data curation, Conceptualization.

## Declaration of competing interest

The authors declare that they have no known competing financial interests or personal relationships that could have appeared to influence the work reported in this paper.

## Figures and Tables

**Supplementary Figure 1:**
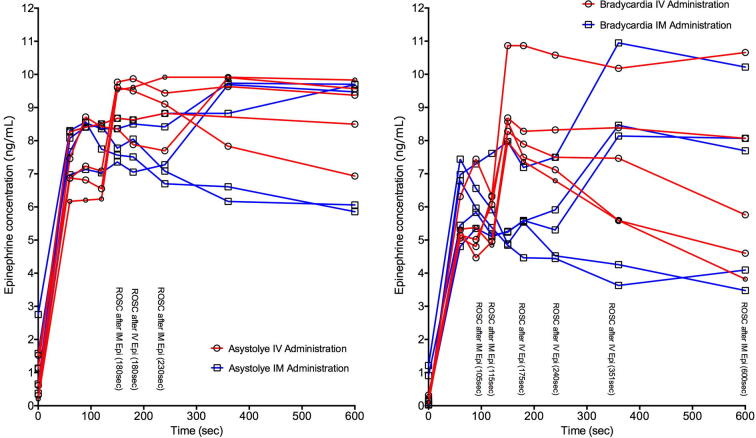
Epinephrine concentration over time plotted for each individual animal within the IM and IV groups with time to ROSC.
